# Objective measurement of retention of laparoscopic skills: a prospective cohort study

**DOI:** 10.1097/JS9.0000000000000272

**Published:** 2023-04-03

**Authors:** A. Masie Rahimi, Sem F. Hardon, Samuel R. Scholten, H. Jaap Bonjer, Freek Daams

**Affiliations:** a Department of Surgery, Amsterdam UMC – VU University Medical Center; b Amsterdam Skills Centre for Health Sciences; c Cancer Center Amsterdam, Amsterdam, The Netherlands

**Keywords:** laparoscopic surgery, laparoscopy training, objective assessment, simulation training, skill decay, skill retention

## Abstract

There has been an overall growth of 462% in laparoscopic procedures performed by surgical residents between 2000 and 2018. Therefore, training courses in laparoscopic surgery are advocated in many postgraduate programs. While the immediate effect is determined in some cases, the retention of acquired skills is rarely investigated. The objective of this study was to objectively measure the retention of laparoscopic technical skills to offer a more personalized training program.

**Methods:**

First year general surgery residents performed two fundamental laparoscopic skills tasks (Post and Sleeve and the ZigZag loop) on the Lapron box trainer. Assessment was performed before, directly after, and 4 months after completing the basic laparoscopy course. Force, motion, and time were the measured variables.

**Results:**

A total of 29 participants were included from 12 Dutch training hospitals and 174 trials were analyzed. The 4 months assessment of the Post and Sleeve showed a significant improvement in force (*P=*0.004), motion (*P*≤0.001), and time (*P*≤0.001) compared to the baseline assessment. The same was true for the ZigZag loop: force (*P*≤0.001), motion (*P=*0.005), and time (*P*≤0.001).

Compared to the 4 months assessment, skill deterioration was present for the Post and Sleeve in the mean force (*P=*0.046), max impulse (*P=*0.12), and time (*P=*0.002). For the ZigZag loop, skill decay was observed for force (*P=*0.021), motion (*P=*0.015), and time (*P*≤0.001) parameters.

**Conclusion:**

Acquired laparoscopic technical skills decreased 4 months after the basic laparoscopy course. Compared to baseline performance, participants showed significant improvement, however deterioration was observed compared to postcourse measurements. To preserve acquired laparoscopic skills, it is recommended to incorporate maintenance training, preferably with objective parameters, in training curricula.

## Introduction

Highlights

- Objective assessment in laparoscopic training, showed skill decay after 4 months.
- This is the first study to assess skill retention with force, motion, and time.
- The trainees remain better compared to their baseline assessment.
- Previous studies used subjective assessment.
- Maintenance laparoscopic simulation training using objective feedback is essential.

The increase of minimally invasive surgery (MIS) is a growing trend across multiple countries and within multiple surgical specialties^1^–^4^. The implementation of MIS has been an essential development in surgery with many benefits such as: faster postoperative recovery, shorter hospital stay, less surgical trauma, and reduced scarring compared to traditional open surgery^5^–^10^. Furthermore, an increase in the demand for enhancing and training laparoscopic technical skills of surgeons and residents has also been observed^11^–^13^. Surgical residents had an increase of 462% in laparoscopic procedures between 2000 and 2018^3^.

Training in MIS techniques such as laparoscopy has proven to reduce errors during surgery and to optimize patient safety and outcomes^14^–^19^. Regardless of training curricula methods such as virtual reality training, video training or laparoscopic box training, assessment of technical skill is also important. Assessment and feedback on performance offer trainees with a clear goal and the degree of skill progression also increases as trainees are more motivated^20^–^22^. Optimization of MIS training, in order to reduce costs of expensive facilities, requires knowledge on effect of training and retention of trained skills.

Prior studies have investigated and observed surgical skill decay using subjective assessment methods and reported the importance of maintenance training^23^,^24^. However, no objective parameters have been priorly used to compare baseline with retention measurements. In other words, a research gap exists regarding defining laparoscopic skills retention and deterioration, using objective parameters such as force and tissue manipulation parameters.

Previously, our research team validated the basic laparoscopy course (BLC) (Amsterdam Skills Centre) using objective force, motion, and time parameters^22^,^25^. The BLC showed improvement of basic technical laparoscopic skills of surgical residents in an early phase of the learning curve. Next to this, studies have shown that maintenance training should be part of every simulator curriculum as surgical skill decay was observed using subjective assessment methods^23^,^24^,^26^–^28^. The aim of this study was to analyze long-term MIS skill retention, based on objective parameters in surgical novices.

## Methods

### Study design and participants

In this multicenter prospective cohort study first year Dutch surgical residents (PGY 1) that completed the BLC were included for the analysis. Ethical Review Board approval was not required and participation was on a voluntary basis. The participants received a questionnaire (Supplemental File A, Supplemental Digital Content 1, http://links.lww.com/JS9/A167) for baseline characteristics, average time since the completion of the BLC and laparoscopic experience.

### Protocol

The BLC consists of a 3 weeks at-home laparoscopic box training with seven previously validated laparoscopic tasks^22^,^29^. The BLC is followed by a training day 4–6 months after the course in which the participants perform a laparoscopic cholecystectomy and appendectomy on fix for life cadaver models^30^.

**Figure 1:**
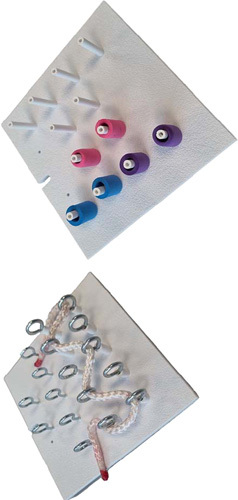
The Post and Sleeve and the ZigZag loop.

**Figure 2:**
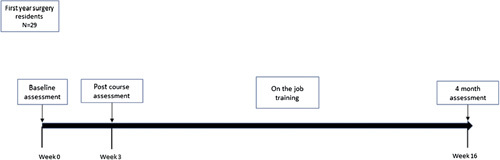
Study protocol.

To assess retention of skill, data of two tasks of different complexity were analyzed (Fig. 1, Post and Sleeve and ZigZag Loop). Participants performed a baseline assessment (BLA) before the BLC, a postcourse assessment (PCA) after the BLC and a retention test at the 4–6 months training day (4MA) (Fig. 2). The objective parameters of the 4MA were compared to the BLA and the PCA. Study protocol and work was reported according to STROCSS criteria^31^, Supplemental Digital Content 2, http://links.lww.com/JS9/A168.

### Subanalysis of the 4MA

Analyses were performed to compare the effect of time between box training and the 4-month assessment. The participants were divided into three groups: 0–3, 4–6, and greater than 7 months. Furthermore, the effect of experience in laparoscopic procedures (0, 1–10, and >10) and simulation training (0–150, 151–240, and >241 min) on the 4MA was also analyzed.

### Systems and hardware

The Lapron box trainer (Amsterdam Skills Centre, Amsterdam, The Netherlands) (File b, Supplemental File A, Supplemental Digital Content 1, http://links.lww.com/JS9/A167) was used during the BLC. The Lapron box trainers were equipped with the ForceSense objective measuring system (MediShield B.V.)^32^. The box trainer provided in total seventeen force, motion, and time parameters of which the most relevant parameters are shown in Table A1 (Supplemental File A, Supplemental Digital Content 1, http://links.lww.com/JS9/A167)^22^,^32^,^33^. The tasks were recorded and uploaded with the objective parameters to an online database. The Lapron box trainers were also equipped with two curved Maryland grasping forceps (Aesculap, B. Braun) for the laparoscopic tasks.

### Statistical analyses

The objective parameters were imported from the online database into IBM SPSS statistics 28 (SPSS Inc.) for statistical analyses. The Shapiro–Wilk test was performed to test for normality and the data was not normally distributed. Nonparametric tests were chosen for statistical analyses. The Wilcoxon signed-rank test was conducted to compare the 4-month assessment with the baseline course assessment and PCA. Mann–Whitney *U*-test was performed to determine the effect of laparoscopic experience (number of procedures and hours of simulation training) and time since BLC completion, on the retainability of laparoscopic technical skills. Differences were considered statistically significant if *P* less than 0.05.

## Results

### Participants

A total of 174 trials performed by 29 surgery residents (PGY 1), from 12 training hospitals in the Netherlands, were included. The participants had completed the BLC between 2019 and 2022. Time between PCA and retention test was 4 months. Baseline characteristics of participants, laparoscopic experience and time since BLC completion are provided in Supplemental file B, Supplemental Digital Content 1, http://links.lww.com/JS9/A167. Cronbach’s *α* of the questionnaire was determined to be 0.751, indicating high-level internal consistency of the Likert scale questionnaire.

### Four-month assessment compared to postcourse assessment

**Figure 3:**
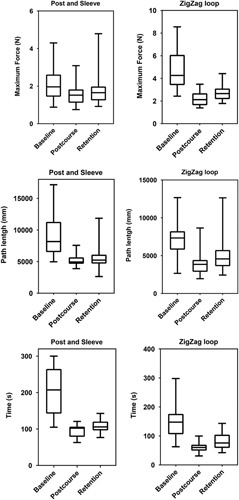
Post and Sleeve and ZigZag loop boxplots of the force, path length, and time measurements.

Table 1 and Figure 3 show the comparison between the 4-month assessment and PCA for both the Post and Sleeve and the ZigZag Loop. For the Post and Sleeve, the max impulse (1.35 vs. 1.04 Ns; *P=*0.012) and mean force (0.22 vs. 0.20 N; *P=*0.046) of the 4MA showed higher forces compared to PCA. Furthermore, there was an increase in time (106 vs. 102 s; *P*=0.002) of the 4MA compared to PCA.

**Table 1:** Four-month assessment (4MA) and postcourse assessment (PCA), presented in median and interquartile range.

Training task	Parameter	Postcourse assessment	Four-month assessment	Difference* (%)	*P*
Post and Sleeve	Penalties (no)	0	0.00 (0.00–0.50)	–	0.347
(*n*=29)	Force volume (N3)	0.06 (0.05–0.09)	0.09 (0.06–0.13)	50	0.161
	Maximum force (N)	1.52 (1.12–1.83)	1.65 (1.25–1.98)	9	0.144
	Maximum impulse (Ns)	1.04 (0.78–1.81)	1.35 (1.03–2.76)	34	*0.012*
	Mean force (N)	0.20 (0.18–0.23)	0.22 (0.18–0.26)	10	*0.046*
	Mean force NZ (N)	0.49 (0.47–0.54)	0.49 (0.45–0.60)	–	0.709
	Total path length (mm)	4930.83 (4644.03–5672.93)	5237.80 (4685.93–6100.05)	6	0.184
	Path length ND (mm)	2452.28 (2259.47–2786.33)	2711.82 (2276.12–3188.60)	11	0.150
	Path length D (mm)	2521.39 (2276.32–2727.58)	2512.12 (2219.60–2936.74)	0	0.871
	SD force (N)	0.19 (0.16–0.24)	0.21 (0.17–0.29)	11	0.334
	Time (s)	102.08 (78.66–106.34)	105.70 (96.24–120.36)	4	*0.002*
ZigZag loop (*n*=28)	Penalties (no)	1.00 (0.00–2.00)	1.50 (1.00–3.75)	50	0.073
	Force volume (N3)	0.21 (0.15–0.30)	0.21 (0.17–0.33)	–	0.414
	Maximum force (N)	2.12 (1.63–2.68)	2.66 (2.17–3.10)	25	*0.021*
	Max impulse	4.55 (3.18–5.34)	5.88 (4.06–6.74)	29	*0.013*
	Mean force (N)	0.44 (0.37–0.51)	0.42 (0.38–0.53)	−5	0.362
	Mean force NZ (N)	0.66 (0.59–0.72)	0.72 (0.66–0.79)	9	0.064
	Total path length (mm)	3849.42 (2865.15–4428.36)	4572.07 (3609.35–5742.16)	19	*0.015*
	Path length ND (mm)	1741.30 (1168.58–2222.23)	2147.09 (1640.72–2860.42)	23	*0.013*
	Path length D (mm)	1897.79 (1547.99–2390.72)	2279.83 (1838.40–2757.20)	20	*0.032*
	SD force (N)	0.37 (0.31–0.45)	0.42 (0.37–0.50)	14	*0.028*
	Time (s)	60.24 (49.54–69.83)	75.74 (58.76–104.39)	26	<*0.001*

* Data from 4-month assessment compared to postcourse assessment.

Italic values are significance of participants.

The 4MA of the ZigZag loop trials had a significant higher max force (2.66 vs. 2.12 N; *P*=0.021), max impulse (5.88 vs. 4.55 Ns; *P*=0.013), and SD Force (0.42 vs. 0.37 N; *P*=0.028) compared to PCA.

Also, the total path length (4572 vs. 3849 mm; *P*=0.015), and time (76 vs. 60 s; *P*≤0.001) were also higher.

### Four-month assessment compared to baseline assessment

The results of the 4MA and BLA comparison are provided in Table 2 and Figure 3. The 4MA of the Post and Sleeve showed significant improvement in max impulse (1.35 vs. 2.19 Ns; *P=*0.004), total path length (8170 vs. 5238 mm; *P*≤0.001) and time (207 vs. 106 s; *P*≤0.001) compared to the BLA.

**Table 2:** Four-month assessment (4MA) and baseline assessment (BLA), presented in median and interquartile range.

Training task	Parameter	Baseline assessment	Four-month assessment	Difference* (%)	*P*
Post and Sleeve	Penalties (no)	0.00 (0.00–2.00)	0.00 (0.00–0.50)	–	0.119
(*n*=29)	Force volume (N3)	0.13 (0.06–0.21)	0.09 (0.06–0.13)	−36	0.171
	Maximum force (N)	1.96 (1.44–2.62)	1.65 (1.25–1.98)	−16	0.084
	Maximum impulse	2.19 (1.32–4.38)	1.35 (1.03–2.76)	−38	*0.004*
	Mean force (N)	0.22 (0.17–0.24)	0.22 (0.18–0.26)	–	0.059
	Mean force NZ (N)	0.55 (0.49–0.66)	0.49 (0.45–0.60)	−11%	0.280
	Total path length (mm)	8170.17 (6501.50–11282.74)	5237.80 (4685.93–6100.05)	−36	<*0.001*
	Path length ND (mm)	4027.30 (3053.55–5879.49)	2711.82 (2276.12–3188.60)	−33	<*0.001*
	Path length D (mm)	4103.24 (3329.01–5592.03)	2512.12 (2219.60–2936.74)	−39	<*0.001*
	SD force (N)	0.22 (0.16–0.33)	0.21 (0.17–0.29)	−5	0.820
	Time (s)	207.28 (142.40–264.88)	105.70 (96.24–120.36)	−49	<*0.001*
ZigZag loop (*n*=28)	Penalties (no)	18.50 (5.00–45.50)	1.50 (1.00–3.75)	−92	<*0.001*
	Force volume (N3)	0.45 (0.30–0.96)	0.21 (0.17–0.33)	−53	<*0.001*
	Maximum force (N)	4.26 (3.41–6.08)	2.66 (2.17–3.10)	−38	<*0.001*
	Maximum impulse	12.21 (8.57–21.20)	5.88 (4.06–6.74)	−52	<*0.001*
	Mean force (N)	0.53 (0.46–0.74)	0.42 (0.38–0.53)	−21	*0.009*
	Mean force NZ (N)	0.81 (0.73–1.02)	0.72 (0.66–0.79)	−11	*0.001*
	Total path length (mm)	7336.25 (5793.35–8222.44)	4572.07 (3609.35–5742.16)	−38	*0.005*
	Path length ND (mm)	3146.91 (2399.47–3999.63)	2147.09 (1640.72–2860.42)	−32	0.092
	Path length D (mm)	3663.92 (3242.76–4300.06)	2279.83 (1838.40–2757.20)	−38	<*0.001*
	SD force (N)	0.55 (0.42–0.74)	0.42 (0.37–0.50)	−24	<*0.001*
	Time (s)	147.62 (105.97–176.01)	75.74 (58.76–104.39)	−49	<*0.001*

* Data from 4-month assessment compared to baseline assessment.

Italic values are significance of participants.

Furthermore, the ZigZag loop 4MA compared to BLA showed improvement for all force parameters: penalties (2 vs. 19; *P*≤0.001), force volume (0.45 vs. 0.21 N^3^; *P*≤0.001), max force (4.26 vs. 2.66 N; *P*≤0.001), max impulse (5.88 vs. 12.21 Ns; *P*≤0.001), mean force (0.53 vs. 0.42 N; *P=*0.009), mean force NZ (0.81 vs. 0.72 N; *P=*0.001) and SD force (0.55 vs. 0.42 N; *P*≤0.001). The same was true for the total path length (7336 vs. 4572 mm; *P*=0.005) and time (148 vs. 76 s; *P*≤0.001).

### Effect of experience in laparoscopic procedures and simulation training

The effect of experience in laparoscopic procedures and simulation training on the 4MA, is presented in Table B5 and B6 (Supplemental File B, Supplemental Digital Content 1, http://links.lww.com/JS9/A167). No significant differences, based on laparoscopic experience, were observed in the objective outcome measurements of the 4MA. Laparoscopic simulation training also showed no significant difference in the 4MA results.

### Effect of time between box training and retention test

The effect of time between the box training and 4MA are demonstrated in Table B4 (Supplemental File B, Supplemental Digital Content 1, http://links.lww.com/JS9/A167). No significant differences were observed in the results of the 4MA of the three groups.

## Discussion

This study demonstrated that participants of the BLC show skill decay, on average after 4 months, for the Post and Sleeve task (3/11 parameters) and ZigZag loop (5/11 parameters) when compared to the PCA. Although, the laparoscopic technical skills of the trainees deteriorate compared to just after the course, the trainees remain better compared to their BLA. For the most difficult task (ZigZag loop), the participants even perform significantly better for all objective parameters (11/11) compared to the first measurement.

No effect was observed on the 4-month assessment regarding laparoscopic experience and time between box training and training day.

Participant performance after 4 months already showed an significant deterioration of laparoscopic technical skill, especially force measurements, and tissue handling parameters. However, this does not exclude that a trainee might have improved in procedural skills or knowledge. Surgical skills simulation training and assessment should not be limited to one-off training courses but be an intrinsic part of the residents’ and surgeons career and learning curve. It is important to define a clear preset proficiency level or expert bench mark to continuously analyze the learning curve of participants during the training years. Specifically, during low volume moments of the residency training program.

Prior research has reported various outcomes regarding the retainability and decay of laparoscopic technical skills. Some studies showed skill retention 1–2 years after the initial measurement^26^–^28^. However, other studies have shown deterioration of laparoscopic technical skills 6 months to 1 year after the baseline test^34^–^38^.

All previously conducted studies used subjective assessment. The studies performed an initial measurement on a laparoscopy box or virtual reality trainer, which was followed up by a postmeasurement at various time intervals. Next to subjective assessment, laparoscopic skills were assessed by total completion time of the task or task specific errors. This does not take into account objective parameters such as force (that mimic tissue manipulation) and the movements of laparoscopic instruments.

A strong point of the current study is the objective measurement of force, motion, and time, avoiding subjective assessment/evaluation by individual assessors. This results in no interrater variability and the assessment can be compared objectively. Also, this study had a relatively big and homogeneous inclusion of surgical residents (PGY 1) from multiple training hospitals in the Netherlands.

In conclusion, the basic laparoscopic course is efficient in acquiring and retaining fundamental laparoscopic technical skills and the improvement of skill is prevalent after 4 months. To maintain the laparoscopic skills, it is desirable to consistently practice laparoscopic skills with objective force, motion, and time feedback.

## Ethical approval

The Basic Laparoscopy Course is part of the surgical residency program, and participating in the study was voluntary (and without consequences). The study was exempt from Ethical Board review. No patient data was used; no ethics approval was required, and the study has thus not been registered with patient research registries or databases.

## Sources of funding

No sources of funding were used.

## Author contribution

M.R.: conception and design, drafting the article, final approval, study design, inclusions, data collection and analysis, manuscript writing and revisions. S.F.H.: conception and design, drafting the article, final approval, study design, inclusions, data collection, manuscript writing and revisions. S.R.S.: conception and design, drafting the article, final approval, inclusions, data collection and analysis, manuscript writing. H.J.B. and F.D.: conception and design, drafting the article, final approval, study design, methods, manuscript writing, manuscript revisions, supervision.

## Conflicts of interest disclosure

All authors declare that they do not have any conflicts of interest or financial ties to disclose.

## Guarantor

A. Masie Rahimi.

Freek Daams.

## Provenance and peer review

Not commissioned, externally peer-reviewed.

## Supplementary Material
