# Supplementary material for: Objective measurement of retention of laparoscopic skills: a prospective cohort study
Source: Int J Surg. 2023 Apr 3;109(4):723–8. doi: 10.1097/JS9.0000000000000272 (PMC10389389; doi:10.1097/JS9.0000000000000272)

**Supplemental file A:**

*Baseline questionnaire*

Questionnaire for surgical residents that participated in our

Basic Laparoscopy Course

**First of all, thank you for helping us by filling in this questionnaire. We ask you to either fill in your own free answer in writing, circle a response or place an “X” in the box next to the response you desire to select.**

Name: ­­­­­­______________________________________ Date: ___________________

1. What is your age?

______ years

2. What is your gender?

| ロ Male | ロ Female | ロ Other |
| --- | --- | --- |

3. Do you have a dominant hand?

| ロ Yes | ロ No |  |
| --- | --- | --- |

If you selected ‘Yes’ to question 3, which hand is your dominant hand?

| ロ Right hand | ロ Left hand |
| --- | --- |

4. Which residency program are you in:

| ロ Rode Kruis Ziekenhuis Beverwijk | ロ Zaans Medisch Centrum |
| --- | --- |
| ロ Spaarne Gasthuis | ロ Dijklander Ziekenhuis |
| ロ NoordWest Ziekenhuisgroep Alkmaar | ロ Amsterdam UMC |
| ロ OLVG | ロ Flevoziekenhuis |
| ロ Gelre ziekenhuizen Apeldoorn | ロ Amphia ziekenhuis Breda |
| ロ Tergooi ziekenhuis | ロ Albert Scheitzer ziekenhuis Dordrecht |
| ロ Haaglanden MC |  |

5. What is your surgical specialty? (Which department of surgery?)

______________________________________________________

6. Which year of your residency program are you currently in? Please circle the desired response:

1 2 3 4

5 6 7 8

7. Did you have any experience with laparoscopic box training prior to our basic laparoscopy training course?

| ロ Yes | ロ No |  |
| --- | --- | --- |

If you selected ‘No’ to question 7: please proceed to question 8.

If you selected ‘Yes’ to question 7: did this experience concern our specific mobile laparoscopic box trainer?

ロ Yes, it was the same mobile laparoscopic box trainer

ロ No, it was a different laparoscopic box trainer

ロ I already had experience with more than one laparoscopic box trainer

Regardless of which box trainer this concerned, how many hours of experience did you, approximately, already have with laparoscopic box training?

ロ 1-5 ロ 5-10 ロ10-15 ロ 15-20 ロ 20-25 ロ 25+

8. Did you have any experience with an alternative form of laparoscopic simulation training (other than box training) prior to our basic laparoscopy training course?

ロ Yes ロ No

If you selected ‘Yes’, how many hours of experience did you, approximately, already have with this alternative form of laparoscopy simulation training?

ロ 1-5 ロ 5-10 ロ10-15 ロ 15-20 ロ 20-25 ロ 25+

9. How many laparoscopic procedures have you assisted on ***prior*** to the laparoscopic training program?

ロ 0 ロ1-5 ロ 5-10 ロ10-15 ロ 15-20 ロ 20+

10. How many laparoscopic procedures have you performed independently ***prior*** to the laparoscopic training program?

ロ 0 ロ1-5 ロ 5-10 ロ10-15 ロ 15-20 ロ 20+

11. How much time did you, approximately, invest in training with our mobile laparoscopic box trainer during your three weeks of at-home training?

________ hours

12. How many laparoscopic procedures have you assisted on ***during*** the laparoscopic training program?

ロ 0 ロ1-5 ロ 5-10 ロ10-15 ロ 15-20 ロ 20+

13. How many laparoscopic procedures in the operating theater did you perform ***during*** the laparoscopy training program?

ロ 0 ロ1-5 ロ 5-10 ロ10-15 ロ 15-20 ロ 20+

14. How many months ago did you complete the laparoscopic training program?

ロ 1 ロ 2 ロ 3 ロ 4 ロ 5 ロ 6

ロ 7 ロ 8 ロ 9 ロ 10 ロ 11 ロ 12

15. How many laparoscopic procedures have you assisted on ***after*** completing the laparoscopic training program?

ロ 0 ロ1-5 ロ 5-10 ロ10-15 ロ 15-20 ロ 20+

16. How many laparoscopic procedures have you performed independently ***after*** completing the laparoscopic training program?

ロ 0 ロ1-5 ロ 5-10 ロ10-15 ロ 15-20 ロ 20+

17. Are you **currently** involved in the **active practice (daily/weekly)** of one or more of the following activities? You can select multiple options:

| ロ Playing (VR) computer- or video games | ロ Actively practicing a musical instrument |
| --- | --- |
| ロ Practicing handcrafting activities (writing, drawing, painting, embroidering, beading, crocheting, sewing,knitting, etcetera) | ロ Eating with chopsticks |
| ロ Other:____________________ |  |

18. Were you **previously** involved in the practice of one or more of the following activities? You can select multiple options:

| ロ Playing (VR) computer- or video games | ロ Actively practicing a musical instrument |
| --- | --- |
| ロ Practicing handcrafting activities (writing, drawing, painting, embroidering, beading, crocheting, sewing,knitting, etcetera) | ロ Eating with chopsticks |
| ロ Other:____________________ |  |

19. With respect to the following practical and technical skills and competences, how well would you rate yourself (precision, depth perception, hand-eye coordination, fine hand motor skills, manual dexterity) on a scale from 1 to 5? (1 = very poor, 5 = excellent)

| Technical Skill | Hand | 1= very poor 2= poor 3= neutral 4 =good 5= excellent | | | | |
| --- | --- | --- | --- | --- | --- | --- |
| Precision | Right | 1 | 2 | 3 | 4 | 5 |
|  | Left | 1 | 2 | 3 | 4 | 5 |
| Depth perception | Right | 1 | 2 | 3 | 4 | 5 |
|  | Left | 1 | 2 | 3 | 4 | 5 |
| Hand-eye coordination | Right | 1 | 2 | 3 | 4 | 5 |
|  | Left | 1 | 2 | 3 | 4 | 5 |
| Fine hand motor skills | Right | 1 | 2 | 3 | 4 | 5 |
|  | Left | 1 | 2 | 3 | 4 | 5 |
| Manual dexterity | Right | 1 | 2 | 3 | 4 | 5 |
|  | Left | 1 | 2 | 3 | 4 | 5 |

Optional: Do you have any comments about your experiences with our basic laparoscopy course?

**Thank-you for your participation in this survey**

**Table A1**

| *Description of the objective ForceSense parameters* | | |
| --- | --- | --- |
| ForceSense parameter | Unit | Description |
| Penalties | Number | The number of penalties imposed if executed forces were above threshold. |
| Force volume | Cubic Newton (N^3^) | The volume of an ellipsoid fitted around the standard deviation of the force along 3 principal components. High force volume indicates fast increasing and decreasing forces in different directions. |
| Maximal force | Newton (N) | The highest absolute force measured during the execution of an attempt at the respective training task (e.g., indicating shaky or jerky movements). |
| Max impulse | Newton seconds (Ns) | The largest product of the force and the duration that the force was exerted, before returned to zero. |
| Mean force | Newton | The average absolute force applied on the task platform. |
| Mean force NZ (non-zero) | Newton | The average absolute force applied on the task platform during periods when the force is not zero. |
| Total path length (mm) | Millimeters (mm) | Total distance travelled by the tip of the right and left instrument during the task. |
| Path length ND (non-dominant hand) | Millimeters (mm) | Distance travelled by the tip of the instrument held by the non-dominant hand during the task. |
| Path length D (dominant hand) | Millimeters (mm) | Distance travelled by the tip of the instrument held by the dominant hand during the task. |
| Standard deviation of force (N) | Newton | The standard deviation of the absolute force. |
| Time (s) | Seconds | Time measured from the beginning of the task until task completion. |

**Fig A1** Lapron box trainer


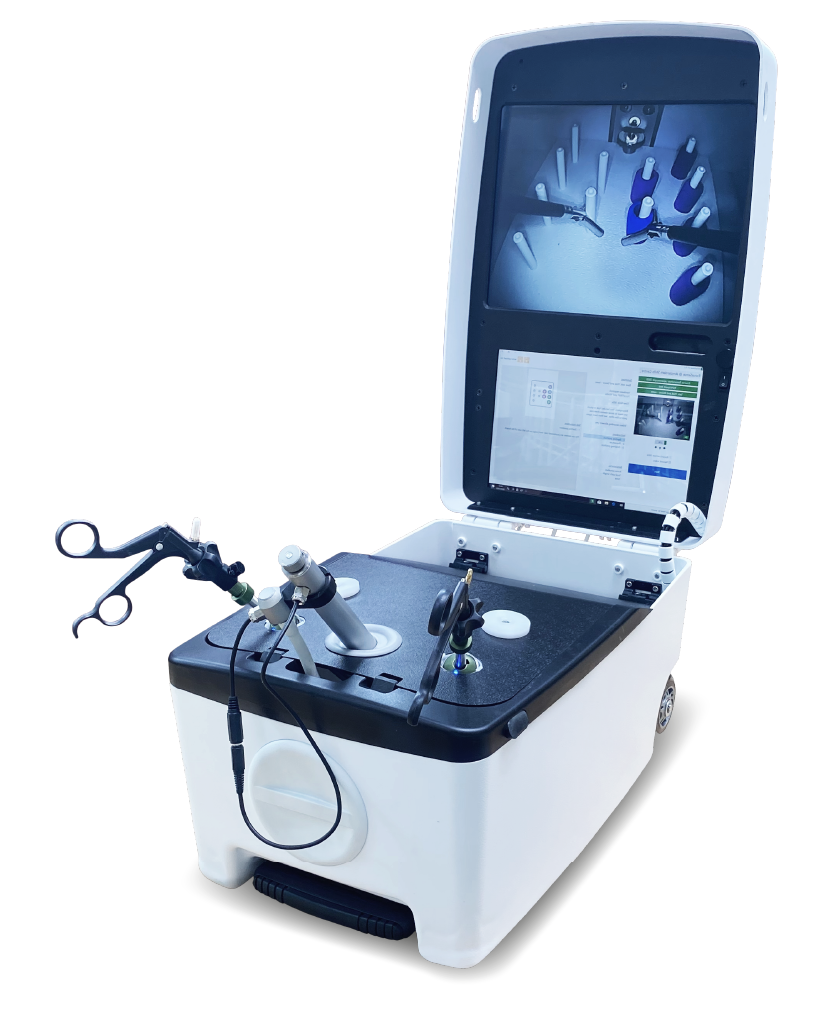


**Supplemental file B:**

**Table B1**

*Baseline characteristics*

| Demographics | Number of residents |
| --- | --- |
| Gender |  |
| Male | 17 |
| Female | 12 |
| Age (years) |  |
| 27 | 1 |
| 28 | 1 |
| 29 | 6 |
| 30 | 7 |
| 31 | 6 |
| 32 | 4 |
| 33 | 2 |
| 36 | 1 |
| 38 | 1 |
| Dominant hand |  |
| Right | 25 |
| Left | 4 |
| Surgical specialty |  |
| General Surgery | 15 |
| Orthopedics/Orthopedic surgery | 8 |
| Plastic Surgery | 2 |
| Urology/Genitourinary surgery | 4 |
| Year in residency program |  |
| First year | 19 |
| Second year | 8 |
| Third year | 2 |
| Experience with laparoscopic box training **prior** to the Basic Laparoscopic Skills Course |  |
| None | 28 |
| With another box trainer (Simendo) | 1 |
| Experience with an alternative form of laparoscopic training **prior** to the Basic Laparoscopic Skills Course |  |
| None | 29 |
| Experience as assisting surgeon in theatre (no. of laparoscopic procedures) |  |
| - **Prior** to the Basic Laparoscopic Skills Course |  |
| None | 1 |
| 1-5 | 2 |
| 5-10 | 6 |
| 10-15 | 4 |
| 15-20 | 3 |
| >20 | 12 |
| - **During and after** the Basic Laparoscopic Skills Course |  |
| None | 3 |
| 1-5 | 5 |
| 5-10 | 4 |
| 10-15 | 2 |
| 15-20 | 1 |
| >20 | 13 |
| Experience as lead surgeon in theatre (no. of laparoscopic procedures) |  |
| - **Prior** to the Basic Laparoscopic Skills Course |  |
| None | 13 |
| 1-5 | 6 |
| 5-10 | 5 |
| 10-15 | 0 |
| 15-20 | 1 |
| >20 | 2 |
| - **During and After** the Basic Laparoscopic Skills Course |  |
| None | 7 |
| 1-5 | 9 |
| 5-10 | 2 |
| 10-15 | 3 |
| 15-20 | 0 |
| >20 | 7 |

**Table B2**

*4 month assessment (4MA) and post course assessment (PCA), presented in median and interquartile range*

| Training task | Parameter | Post-course assessment | Retention test | Difference ^a^ | *p-*value |
| --- | --- | --- | --- | --- | --- |
| Post and Sleeve | Penalties (no) | 0.00  (0.00-0.00) | 0.00  (0.00-0.50) | - | 0.347 |
| (n=29) | Force volume (N^3^) | 0.06  (0.05-0.09) | 0.09  (0.06-0.13) | 50% | 0.161 |
|  | Max Force (N) | 1.52  (1.12-1.83) | 1.65  (1.25-1.98) | 9% | 0.144 |
|  | Max impulse (Ns) | 1.04  (0.78-1.81) | 1.35  (1.03-2.76) | 34% | ***0.012*** |
|  | Mean force (N) | 0.20  (0.18-0.23) | 0.22  (0.18-0.26) | 10% | ***0.046*** |
|  | Mean force NZ (N) | 0.49  (0.47-0.54) | 0.49  (0.45-0.60) | - | 0.709 |
|  | Total path length (mm) | 4930.83  (4644.03-5672.93) | 5237.80  (4685.93-6100.05) | 6% | 0.184 |
|  | Path length ND (mm) | 2452.28  (2259.47-2786.33) | 2711.82  (2276.12-3188.60) | 11% | 0.150 |
|  | Path length D (mm) | 2521.39  (2276.32-2727.58) | 2512.12  (2219.60-2936.74) | 0% | 0.871 |
|  | SD Force (N) | 0.19  (0.16-0.24) | 0.21  (0.17-0.29) | 11% | 0.334 |
|  | Time (s) | 102.08  (78.66-106.34) | 105.70  (96.24-120.36) | 4% | ***0.002*** |
| ZigZag loop  (n=28) | Penalties (no) | 1.00  (0.00-2.00) | 1.50  (1.00-3.75) | 50% | 0.073 |
|  | Force volume (N^3^) | 0.21  (0.15-0.30) | 0.21  (0.17-0.33) | - | 0.414 |
|  | Max Force (N) | 2.12  (1.63-2.68) | 2.66  (2.17-3.10) | 25% | ***0.021*** |
|  | Max impulse (Ns) | 4.55  (3.18-5.34) | 5.88  (4.06-6.74) | 29% | ***0.013*** |
|  | Mean force (N) | 0.44  (0.37-0.51) | 0.42  (0.38-0.53) | -5% | 0.362 |
|  | Mean force NZ (N) | 0.66  (0.59-0.72) | 0.72  (0.66-0.79) | 9% | 0.064 |
|  | Total path length (mm) | 3849.42  (2865.15-4428.36) | 4572.07  (3609.35-5742.16) | 19% | ***0.015*** |
|  | Path length ND (mm) | 1741.30  (1168.58-2222.23) | 2147.09  (1640.72-2860.42) | 23% | ***0.013*** |
|  | Path length D (mm) | 1897.79  (1547.99-2390.72) | 2279.83  (1838.40-2757.20) | 20% | ***0.032*** |
|  | SD Force (N) | 0.37  (0.31-0.45) | 0.42  (0.37-0.50) | 14% | ***0.028*** |
|  | Time (s) | 60.24  (49.54-69.83) | 75.74  (58.76-104.39) | 26% | ***<0.001*** |
| ^a^Data from retention test compared to post-course assessment | | | | | |

**Table B3**

*4 month assessment (4MA) and baseline assessment (BLA), presented in median and interquartile range*

| Training task | Parameter | Baseline assessment | Retention test | Difference^a^ | *p-*value |
| --- | --- | --- | --- | --- | --- |
| Post and Sleeve | Penalties (no) | 0.00  (0.00-2.00) | 0.00  (0.00-0.50) | - | 0.119 |
| (n=29) | Force volume (N^3^) | 0.13  (0.06-0.21) | 0.09  (0.06-0.13) | -36% | 0.171 |
|  | Max Force (N) | 1.96  (1.44-2.62) | 1.65  (1.25-1.98) | -16% | 0.084 |
|  | Max impulse | 2.19  (1.32-4.38) | 1.35  (1.03-2.76) | -38% | ***0.004*** |
|  | Mean force (N) | 0.22  (0.17-0.24) | 0.22  (0.18-0.26) | - | 0.059 |
|  | Mean force NZ (N) | 0.55  (0.49-0.66) | 0.49  (0.45-0.60) | -11% | 0.280 |
|  | Total path length (mm) | 8170.17  (6501.50-11282.74) | 5237.80  (4685.93-6100.05) | -36% | ***<0.001*** |
|  | Path length ND (mm) | 4027.30  (3053.55-5879.49) | 2711.82  (2276.12-3188.60) | -33% | ***<0.001*** |
|  | Path length D (mm) | 4103.24  (3329.01-5592.03) | 2512.12  (2219.60-2936.74) | -39% | ***<0.001*** |
|  | SD Force (N) | 0.22  (0.16-0.33) | 0.21  (0.17-0.29) | -5% | 0.820 |
|  | Time (s) | 207.28  (142.40-264.88) | 105.70  (96.24-120.36) | -49% | ***<0.001*** |
| ZigZag loop  (n=28) | Penalties (no) | 18.50  (5.00-45.50) | 1.50  (1.00-3.75) | -92% | ***<0.001*** |
|  | Force volume (N^3^) | 0.45  (0.30-0.96) | 0.21  (0.17-0.33) | -53% | ***<0.001*** |
|  | Max Force (N) | 4.26  (3.41-6.08) | 2.66  (2.17-3.10) | -38% | ***<0.001*** |
|  | Max impulse | 12.21  (8.57-21.20) | 5.88  (4.06-6.74) | -52% | ***<0.001*** |
|  | Mean force (N) | 0.53  (0.46-0.74) | 0.42  (0.38-0.53) | -21% | ***0.009*** |
|  | Mean force NZ (N) | 0.81  (0.73-1.02) | 0.72  (0.66-0.79) | -11% | ***0.001*** |
|  | Total path length (mm) | 7336.25  (5793.35-8222.44) | 4572.07  (3609.35-5742.16) | -38% | ***0.005*** |
|  | Path length ND (mm) | 3146.91  (2399.47-3999.63) | 2147.09  (1640.72-2860.42) | -32% | 0.092 |
|  | Path length D (mm) | 3663.92  (3242.76-4300.06) | 2279.83  (1838.40-2757.20) | -38% | ***<0.001*** |
|  | SD Force (N) | 0.55  (0.42-0.74) | 0.42  (0.37-0.50) | -24% | ***<0.001*** |
|  | Time (s) | 147.62  (105.97-176.01) | 75.74  (58.76-104.39) | -49% | ***<0.001*** |
| ^a^Data from retention test compared to baseline assessment | | | | | |

**Table B4**

*Results of the 4 month assessment, displayed in three groups with different time interval since course completion. Parameters outcomes presented in median and interquartile range.*

| Training task | Parameter | Group A  0-3 months  (n=14) | Group B  4-6 months  (n=10) | Group C  >7 months  (n=4) | *p*-value  A-B | *p*-value  A-C | *p*-value  B-C |
| --- | --- | --- | --- | --- | --- | --- | --- |
| Post and Sleeve | Penalties (no) | 0.00  (0.00-1.00) | 0.00  (0.00-0.25) | 0.00  (0.00-0.00) | 0.892 | 0.469 | 0.454 |
|  | Force volume (N^3^) | 0.08  (0.05-0.13) | 0.09  (0.06-0.14) | 0.11  (0.06-0.20) | 0.807 | 0.530 | 0.939 |
|  | Max Force (N) | 1.57  (1.28-2.01) | 1.68  (1.21-2.15) | 1.80  (1.31-1.93) | 0.807 | 0.665 | 1.000 |
|  | Max impulse (Ns) | 1.68  (0.97-3.22) | 1.31  (1.06-2.39) | 1.86  (0.83-2.97) | 0.643 | 0.810 | 0.733 |
|  | Mean force (N) | 0.22  (0.18-0.26) | 0.23  (0.20-0.29) | 0.21  (0.15-0.25) | 0.461 | 0.665 | 0.304 |
|  | Mean force NZ (N) | 0.49  (0.45-0.58) | 0.51  (0.46-0.66) | 0.53  (0.45-0.65) | 0.683 | 0.810 | 0.945 |
|  | Total path length (mm) | 5004.46  (4620.28-5999.73) | 5663.43  (4422.54-6237.65) | 5414.95  (4882.43-5785.21) | 0.495 | 0.596 | 0.839 |
|  | Path length ND (mm) | 2511.75  (2153.38-2962.47) | 2973.79  (2295.47-3390.85) | 2669.69  (2507.61-3276.69) | 0.261 | 0.596 | 0.945 |
|  | Path length D (mm) | 2512.24  (2108.54-2994.79) | 2498.49  (2127.06-3092.52) | 2522.33  (2312.16-2794.23) | 0.807 | 1.000 | 0.945 |
|  | SD Force (N) | 0.20  (0.17-0.28) | 0.22  (0.17-0.31) | 0.22  (0.16-0.30) | 0.723 | 0.961 | 0.945 |
|  | Time (s) | 103.66  (87.64-115.28) | 114.65  (96.92-123.44) | 102.47  (97.01-118.64) | 0.160 | 0.736 | 0.539 |
| ZigZag loop | Penalties (no) | 1.50  (1.00-5.75) | 1.50  (0.00-11.50) | 1.50  (0.25-3.50) | 0.796 | 0.645 | 0.945 |
|  | Force volume (N^3^) | 0.22  (0.16-0.38) | 0.21  (0.17-0.23) | 0.22  (0.18-0.31) | 0.709 | 0.721 | 0.839 |
|  | Max Force (N) | 2.9  (2.33-3.26) | 2.33  (1.97-3.31) | 2.60  (2.01-2.88) | 0.285 | 0.233 | 1.000 |
|  | Max impulse (Ns) | 5.42  (3.82-6.75) | 5.40  (3.73-7.03) | 6.65  (6.40-7.70) | 1.000 | 0.127 | 0.106 |
|  | Mean force (N) | 0.44  (0.38-0.51) | 0.40  (0.32-0.57) | 0.48  (0.40-0.57) | 0.472 | 0.654 | 0.374 |
|  | Mean force NZ (N) | 0.71  (0.67-0.79) | 0.71  (0.59-0.80) | 0.73  (0.68-0.73) | 0.666 | 0.798 | 0.839 |
|  | Total path length (mm) | 3687.58  (3086.44-6197.84) | 4938.34  (4258.57-5763.08) | 4572.07  (4136.33-5506.82) | 0.472 | 0.574 | 0.733 |
|  | Path length ND (mm) | 1991.64  (1472.45-3196.78) | 2187.75  (1924.41-2674.61) | 2176.72  (2047.33-3266.55) | 0.585 | 0.574 | 0.839 |
|  | Path length D (mm) | 2169.19  (1554.31-2802.36) | 2451.42  (2291.59-3029.23) | 2186.50  (2063.27-2474.44) | 0.154 | 0.959 | 0.142 |
|  | SD Force (N) | 0.43  (0.39-0.52) | 0.39  (0.33-0.50) | 0.43  (0.38-0.47) | 0.403 | 0.721 | 0.635 |
|  | Time (s) | 65.15  (53.53-88.96) | 94.55  (72.41-108.11) | 73.93  (62.27-99.30) | 0.084 | 0.505 | 0.374 |
|  | | | | | | | |

**Table B5**

*Results of 4 month assessment, displayed in three groups with different amount of training during initial program. Parameters outcomes presented in median and interquartile range.*

| Training task | Parameter | Group A  0-150 min  (n=7) | Group B  151-240 min  (n=11) | Group C  >241 min  (n=10 | *p*-value  A-B | *p*-value  A-C | *p*-value  B-C |
| --- | --- | --- | --- | --- | --- | --- | --- |
| Post and Sleeve | Penalties (no.) | 0.00  (0.00-1.00) | 0.00  (0.00-0.00) | 0.00  (0.00-0.75) | 0.657 | 0.762 | 0.918 |
|  | Force volume (N^3^) | 0.11  (0.05-0.12) | 0.08  (0.05-0.13) | 0.09  (0.08-0.18) | 0.840 | 0.515 | 0.387 |
|  | Max Force (N) | 1.85  (1.67-2.04) | 1.28  (1.19-1.68) | 1.65  (1.45-2.05) | 0.152 | 0.573 | 0.152 |
|  | Max impulse (Ns) | 2.12  (1.21-3.03) | 1.18  (0.75-2.08) | 1.80  (1.19-3.44) | 0.177 | 0.965 | 0.152 |
|  | Mean force (N) | 0.23  (0.19-0.26) | 0.20  (0.18-0.28) | 0.23  (0.18-0.27) | 0.657 | 1.000 | 0.605 |
|  | Mean force NZ (N) | 0.51  (0.46-0.64) | 0.46  (0.44-0.56) | 0.57  (0.48-0.62) | 0.272 | 0.573 | 0.085 |
|  | Total path length (mm) | 5548.01  (4325.18-6231.79) | 5274.99  (4253.85-6308.84) | 5126.70  (4788.42-5631.68) | 1.000 | 0.633 | 0.756 |
|  | Path length ND (mm) | 2922.62  (2108.88-2976.74) | 2801.65  (2211.53-3760.91) | 2529.74  (2299.78-3198.53) | 1.000 | 1.000 | 0.605 |
|  | Path length D (mm) | 2695.47  (2301.67-3234.03) | 2573.61  (2042.32-3041.35) | 2432.70  (2278.73-2751.37) | 0.492 | 0.408 | 0.973 |
|  | SD Force (N) | 0.23  (0.17-0.29) | 0.18  (0.16-0.22) | 0.22  (0.20-0.31) | 0.395 | 0.829 | 0.132 |
|  | Time (s) | 108.63  (98.86-116.74) | 103.34  (87.64-132.44) | 107.02  (93.82-122.03) | 0.717 | 0.897 | 0.973 |
| ZigZag loop | Penalties (no.) | 1.00  (1.00-8.00) | 2.00  (1.00-3.00) | 1.50  (0.00-5.25) | 0.930 | 0.813 | 0.863 |
|  | Force volume (N^3^) | 0.21  (0.17-0.32) | 0.20  (0.17-0.39) | 0.23  (0.19-0.36) | 1.000 | 0.475 | 0.605 |
|  | Max Force (N) | 2.29  (2.15-3.11) | 2.63  (2.36-2.99) | 2.92  (2.00-3.27) | 0.375 | 0.601 | 0.512 |
|  | Max impulse (Ns) | 7.06  (6.00-8.81) | 5.82  (3.97-6.65) | 5.02  (3.45-6.40) | 0.151 | 0.161 | 0.557 |
|  | Mean force (N) | 0.41  (0.37-0.52) | 0.42  (0.41-0.52) | 0.42  (0.37-0.56) | 0.659 | 0.887 | 0.654 |
|  | Mean force NZ (N) | 0.74  (0.69-0.79) | 0.69  (0.66-0.73) | 0.73  (0.60-0.80) | 0.211 | 0.740 | 0.512 |
|  | Total path length (mm) | 4279.32  (4045.12-5514.50) | 4280.11  (3040.01-5633.64) | 5350.55  (3714.67-7245.84) | 0.596 | 0.475 | 0.152 |
|  | Path length ND (mm) | 2121.39  (1975.96-2604.17) | 2237.15  (1588.21-2476.27) | 2265.74  (1781.57-4451.80) | 0.536 | 0.962 | 0.387 |
|  | Path length D (mm) | 2303.36  (2039.07-2910.33) | 2237.15  (1588.21-2476.27) | 2569.72  (2044.77-2514.66) | 0.425 | 0.475 | 0.173 |
|  | SD Force (N) | 0.45  (0.41-0.53) | 0.39  (0.37-0.44) | 0.45  (0.33-0.50) | 0.179 | 0.962 | 0.459 |
|  | Time (s) | 68.28  (59.64-107.90) | 70.16  (54.24-94.56) | 92.86  (68.11-107.06) | 0.724 | 0.536 | 0.197 |
|  | | | | | | | |

**Table B6**

| *Results of the 4 month assessment, displayed in three groups with different amount of laparoscopic procedures since course completion. Parameters outcomes presented in median and interquartile range.* | | | | | | | |
| --- | --- | --- | --- | --- | --- | --- | --- |
| Training task | Parameter | Group A  0 procedures  (n=8) | Group B  1-10 procedures  (n=10) | Group C  >10 procedures  (n=10) | *p*-value  A-B | *p*-value  A-C | *p*-value  B-C |
| Post and Sleeve | Penalties (no) | 0.00  (0.00-0.75) | 0.00  (0.00-3.00) | 0.00  (0.00-1.00) | 1.000 | 0.965 | 0.912 |
|  | Force volume (N^3^) | 0.08  (0.05-0.11) | 0.09  (0.07-0.14) | 0.10  (0.06-0.15) | 0.315 | 0.460 | 0.971 |
|  | Max Force (N) | 1.60  (1.28-1.97) | 1.57  (1.21-2.04) | 1.87  (1.19-2.03) | 0.965 | 0.762 | 0.796 |
|  | Max impulse (Ns) | 1.33  (1.05-2.93) | 1.47  (0.87-2.77) | 1.59  (1.06-3.08) | 0.965 | 0.762 | 0.853 |
|  | Mean force (N) | 0.23  (0.22-0.28) | 0.20  (0.18-0.26) | 0.22  (0.18-0.28) | 0.360 | 0.515 | 0.631 |
|  | Mean force NZ (N) | 0.46  (0.44-0.54) | 0.53  (0.45-0.60) | 0.53  (0.48-0.61) | 0.408 | 0.173 | 0.739 |
|  | Total path length (mm) | 4701.01  (3313.65-5809.25) | 5236.55  (4872.64-5973.70) | 5414.95  (4683.73-6209.72) | 0.274 | 0.237 | 1.000 |
|  | Path length ND (mm) | 2182.46  (1227.02-3303.89) | 2835.65  (2417.16-3316.86) | 2669.59  (2431.63-3174.99) | 0.146 | 0.173 | 0.796 |
|  | Path length D (mm) | 2337.45  (1970.07-2874.15) | 2521.00  (2296.33-2800.93) | 2602.58  (2251.74-2977.84) | 0.274 | 0.274 | 0.739 |
|  | SD Force (N) | 0.20  (0.17-0.28) | 0.21  (0.17-0.31) | 0.22  (0.16-0.27) | 0.460 | 0.460 | 0.971 |
|  | Time (s) | 103.91  (83.19-113.37) | 104.87  (93.90-122.03) | 108.13  (98.03-125.15) | 0.360 | 0.274 | 0.631 |
| ZigZag loop | Penalties (no) | 3.50  (1.00-8.75) | 1.50  (0.00-3.25) | 1.00  (0.00-2.00) | 0.237 | 0.114 | 0.604 |
|  | Force volume (N^3^) | 0.20  (0.15-0.35) | 0.23  (0.19-0.38) | 0.21  (0.18-0.29) | 0.515 | 0.888 | 0.780 |
|  | Max Force (N) | 3.01  (2.45-3.77) | 2.77  (1.98-3.17) | 2.35  (1.94-2.99) | 0.274 | 0.114 | 0.400 |
|  | Max impulse (Ns) | 5.74  (4.87-8.37) | 4.51  (2.46-6.43) | 6.24  (3.94-7.39) | 0.237 | 0.963 | 0.549 |
|  | Mean force (N) | 0.49  (0.39-0.66) | 0.44  (0.39-0.54) | 0.41  (0.36-0.74) | 0.633 | 0.370 | 0.549 |
|  | Mean force NZ (N) | 0.73  (0.69-0.80) | 0.72  (0.65-0.80) | 0.71  (0.59-0.74) | 0.696 | 0.321 | 0.780 |
|  | Total path length (mm) | 5227.22  (3580.83-6204.89) | 3695.58  (3010.14-6688.82) | 4936.73  (4238.22-5699.00) | 0.515 | 0.888 | 0.211 |
|  | Path length ND (mm) | 2486.45  (1628.44-3613.24) | 1729.80  (1454.66-3949.96) | 2172.78  (2014.94-2811.62) | 0.696 | 0.606 | 0.278 |
|  | Path length D (mm) | 2335.50  (1725.46-3304.94) | 1986.33  (1506.22-2738.87) | 2426.57  (2186.50-2793.31) | 0.360 | 0.743 | 0.243 |
|  | SD Force (N) | 0.46  (0.40-0.53) | 0.42  (0.37-0.49) | 0.38  (0.33-0.45) | 0.315 | 0.139 | 0.356 |
|  | Time (s) | 76.59  (56.29-99.37) | 66.07  (45.44-92.70) | 93.56  (69.22-107.20) | 0.408 | 0.277 | 0.156 |
|  | | | | | | | |

**Graph B1**

*Post and Sleeve box plots*


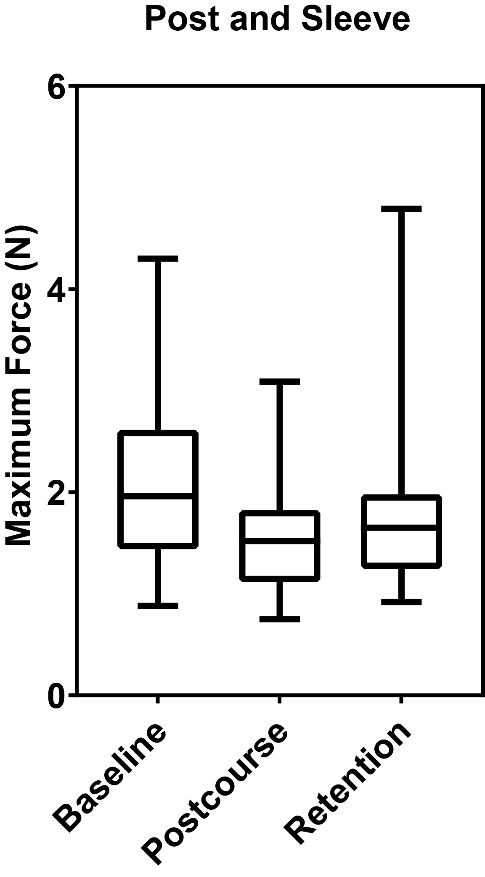


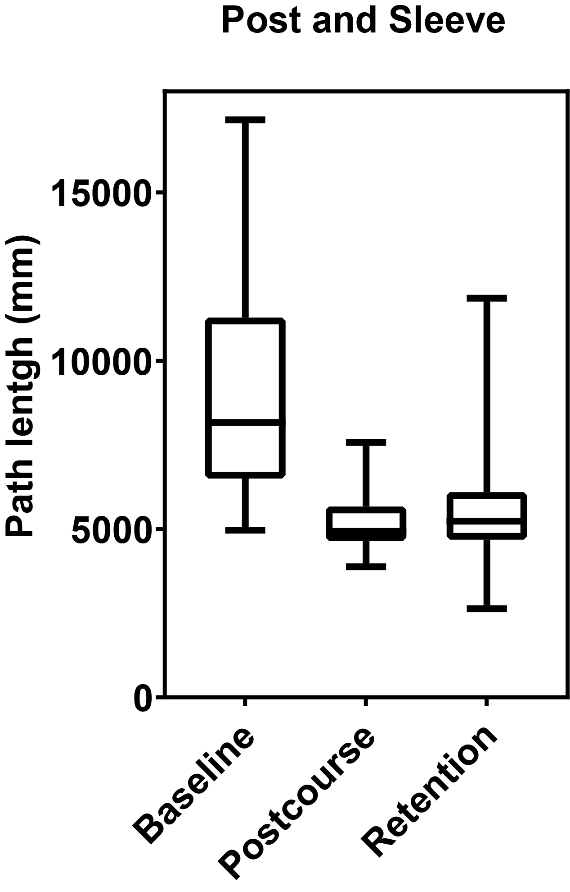

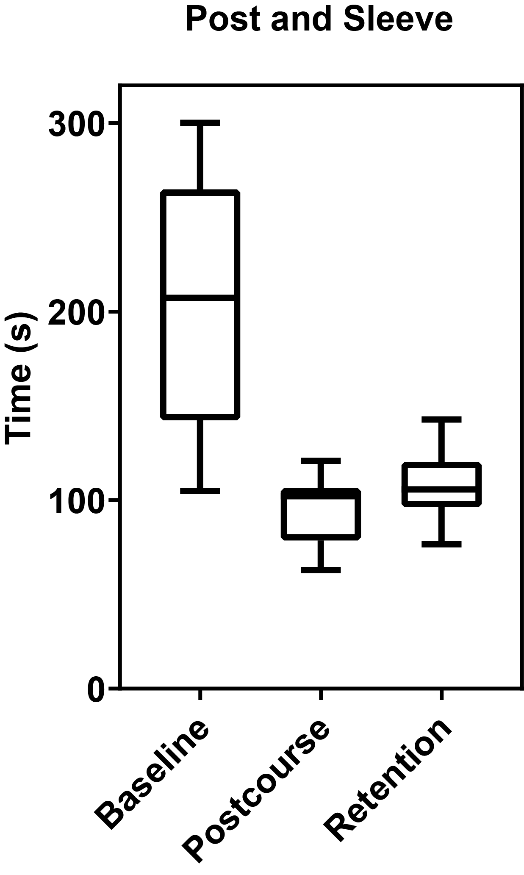


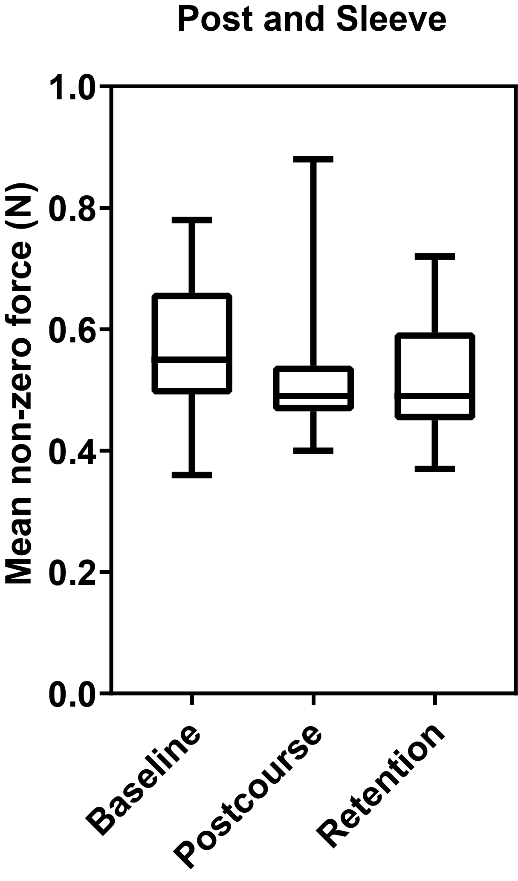

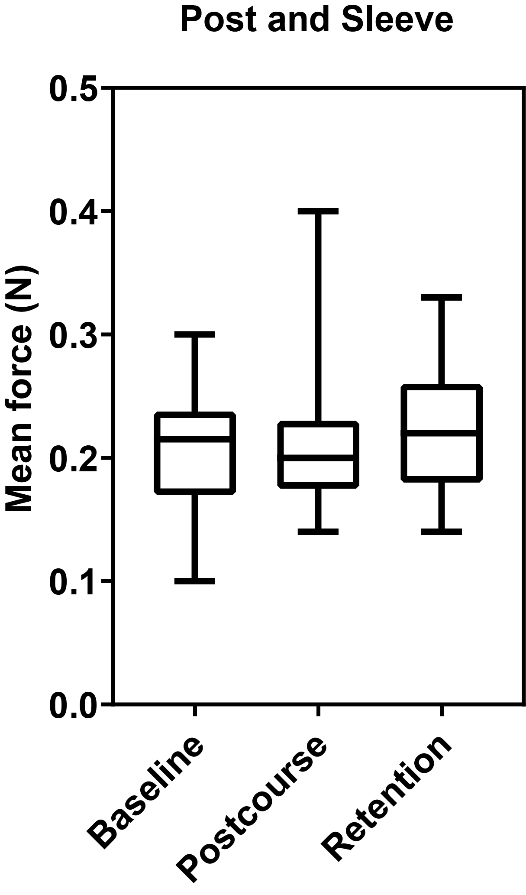

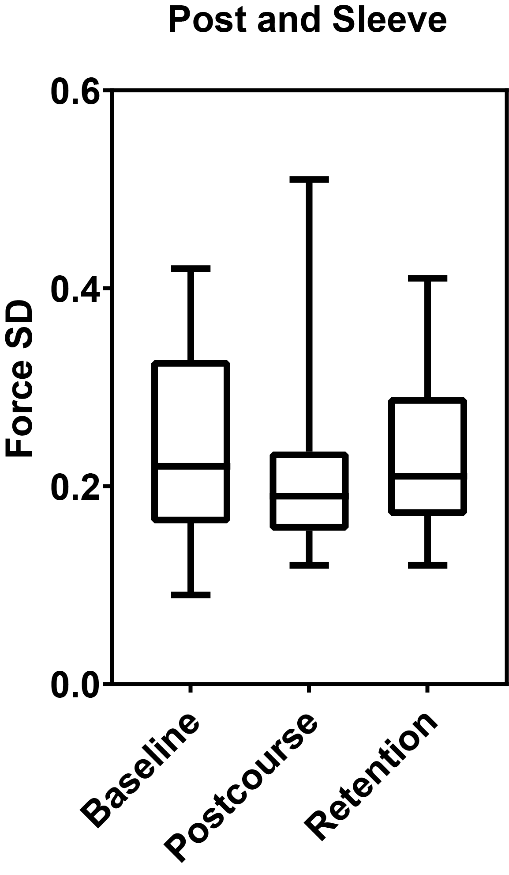


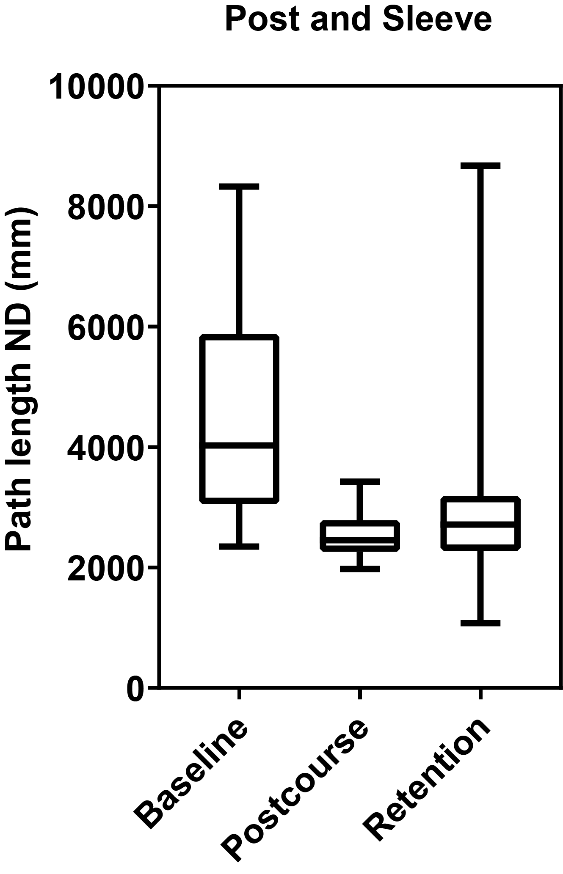

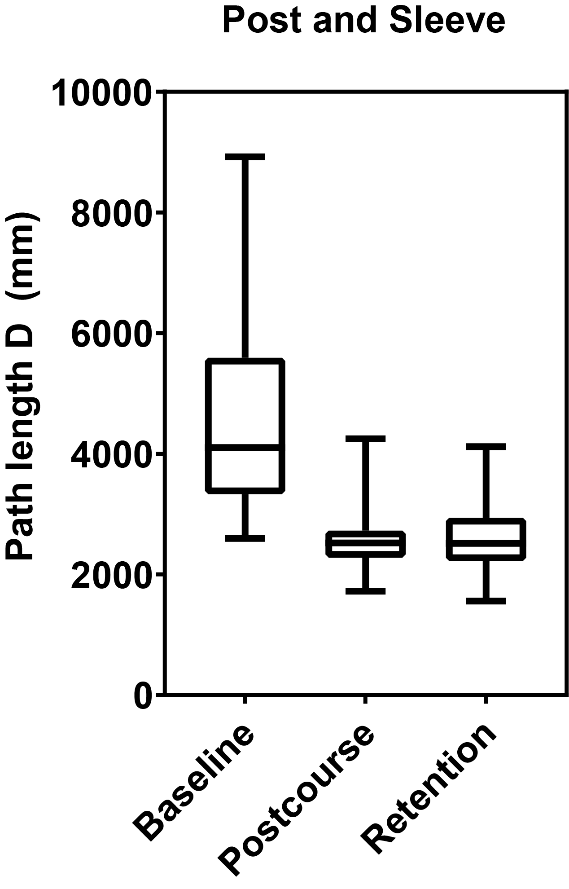

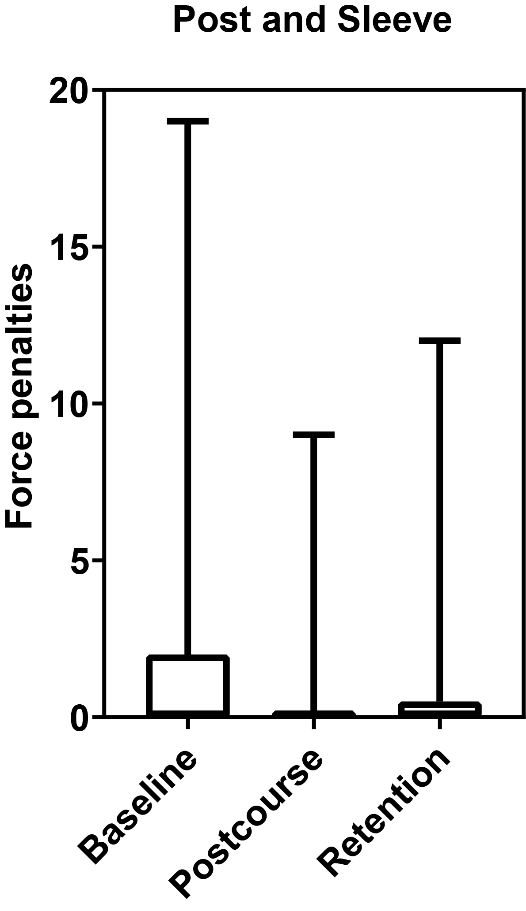

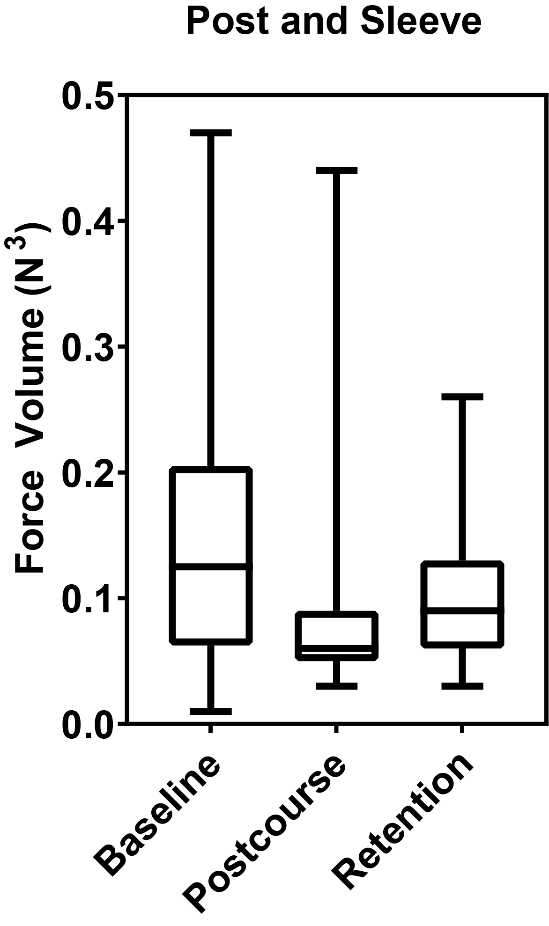

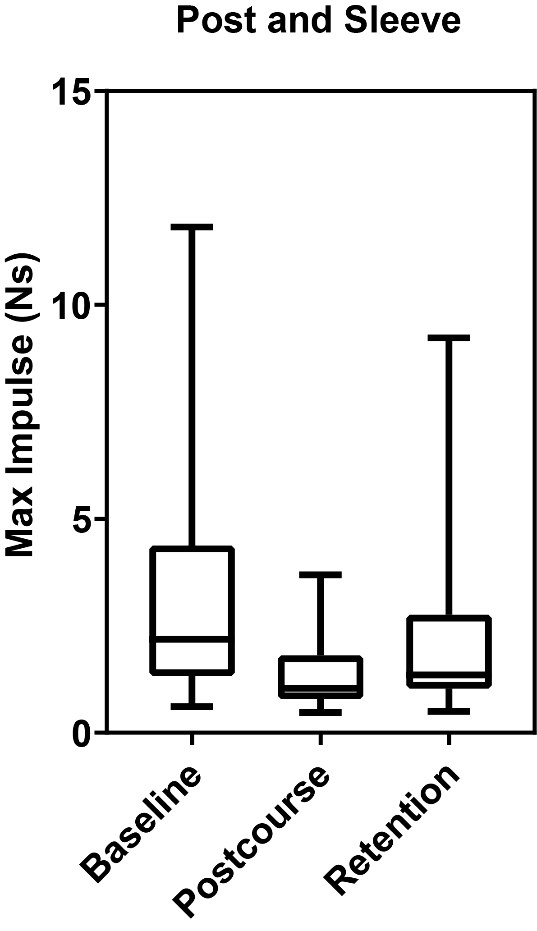


**Graph B2**

*ZigZag loop box plots*


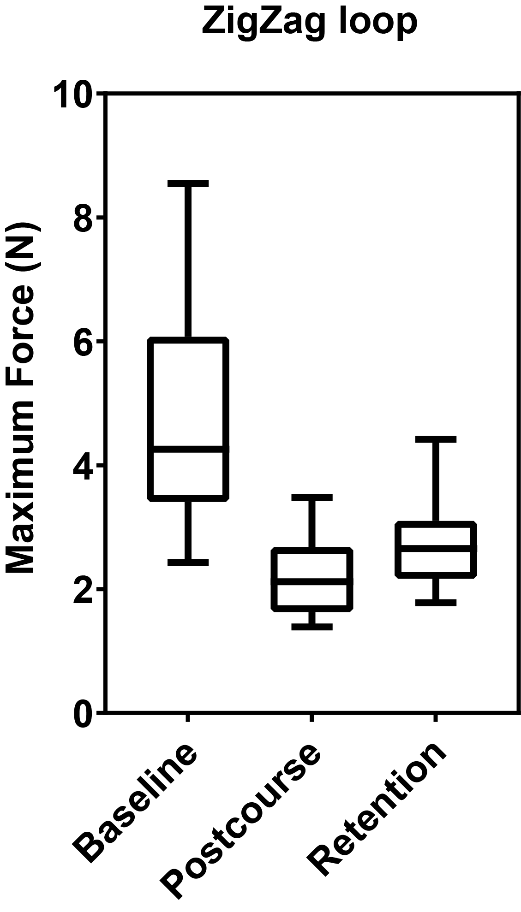


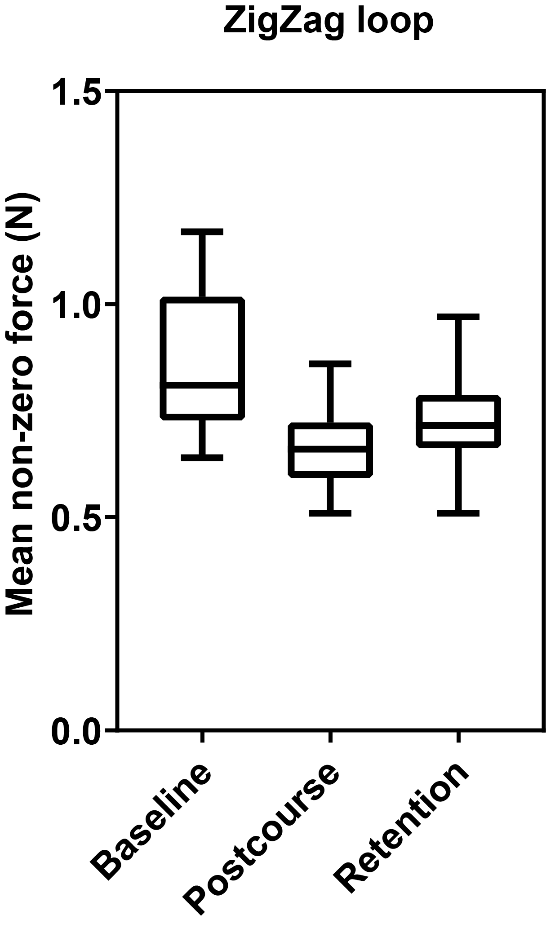

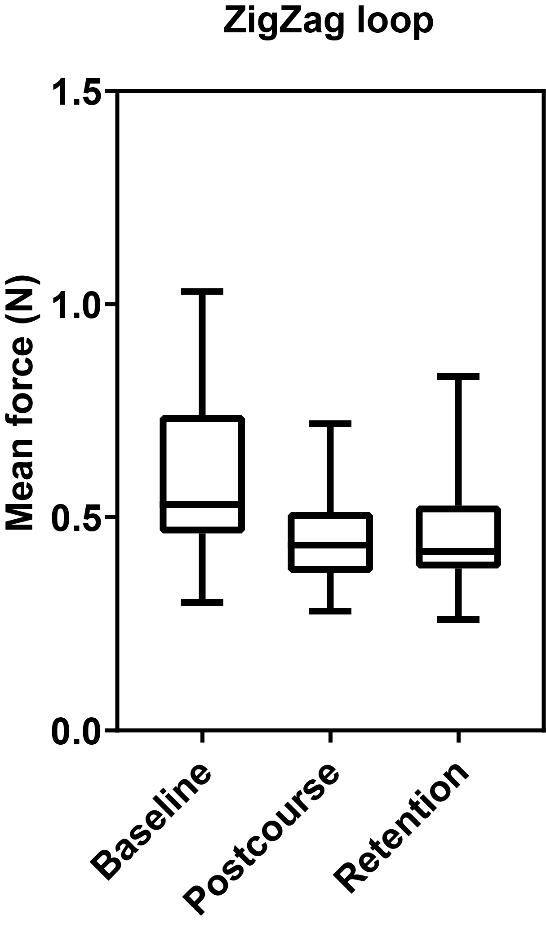

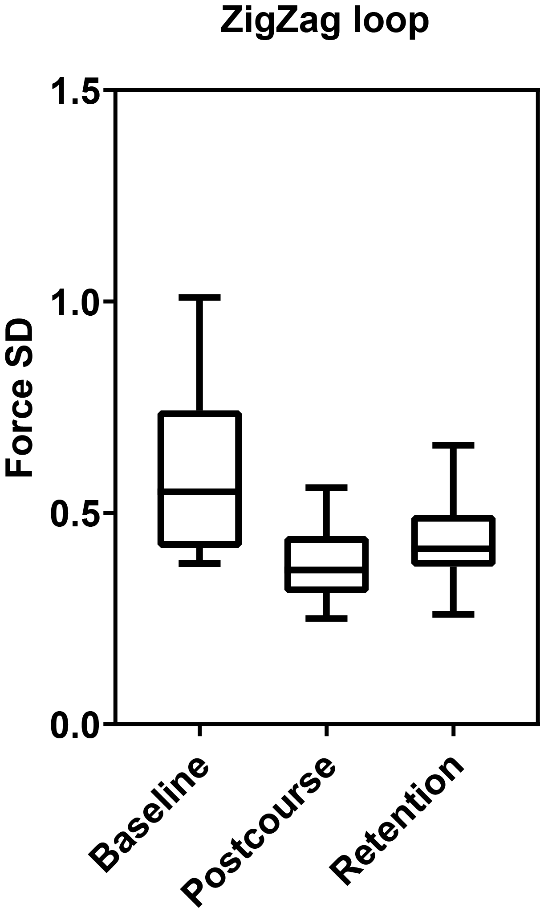

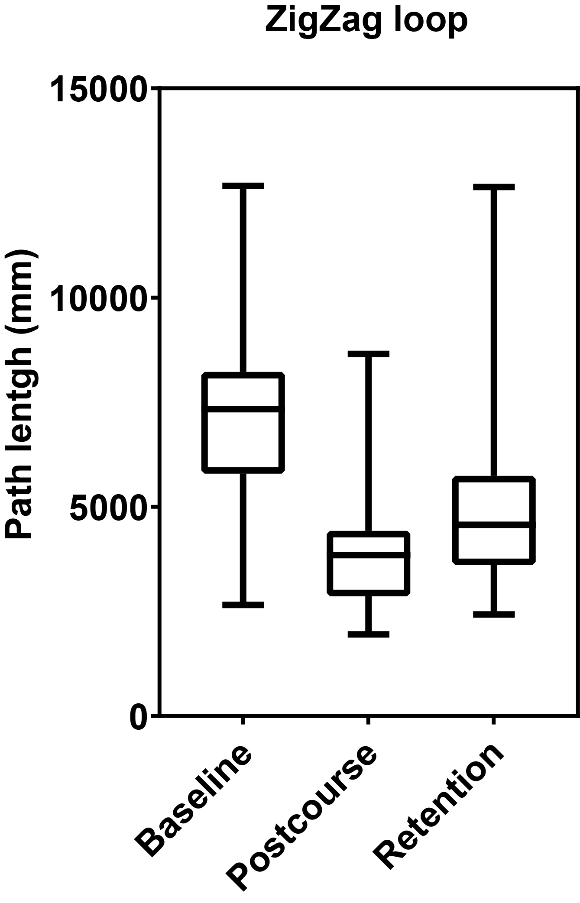

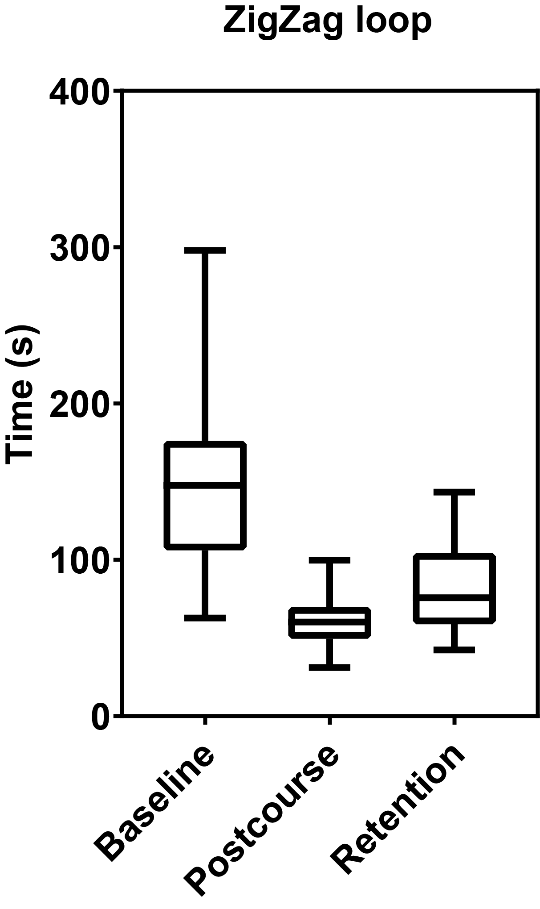


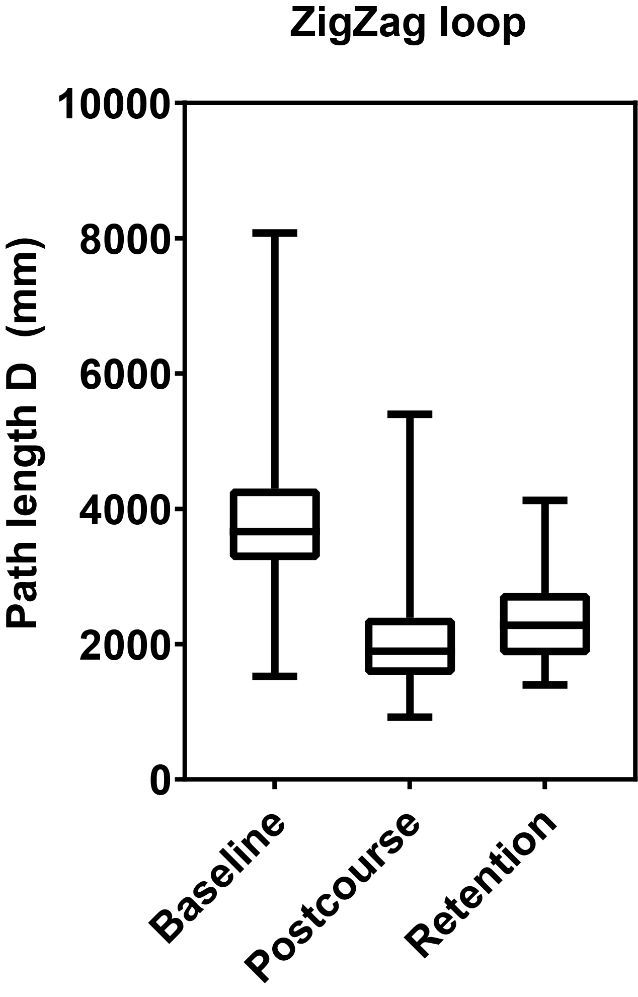

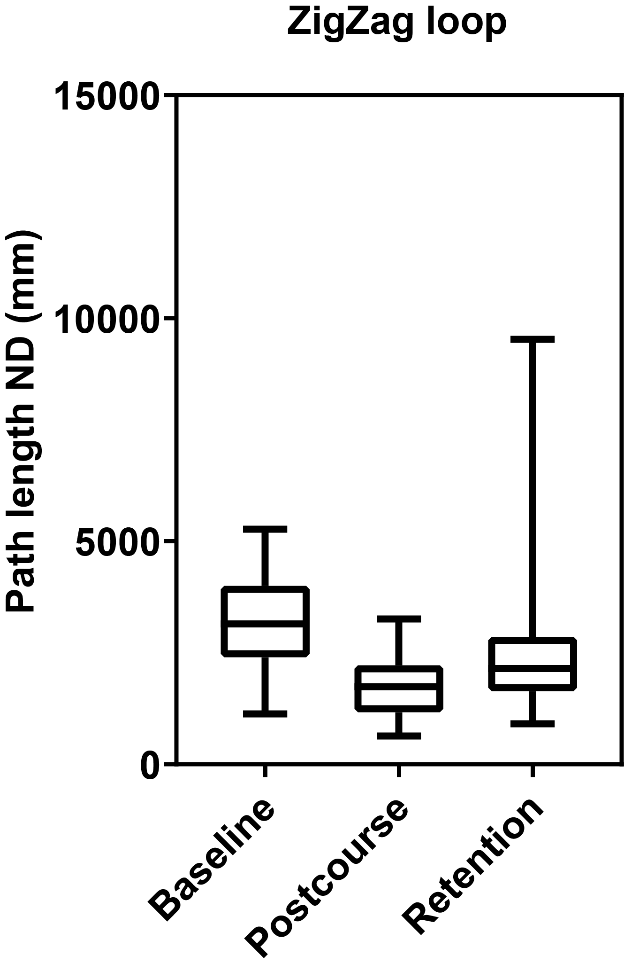

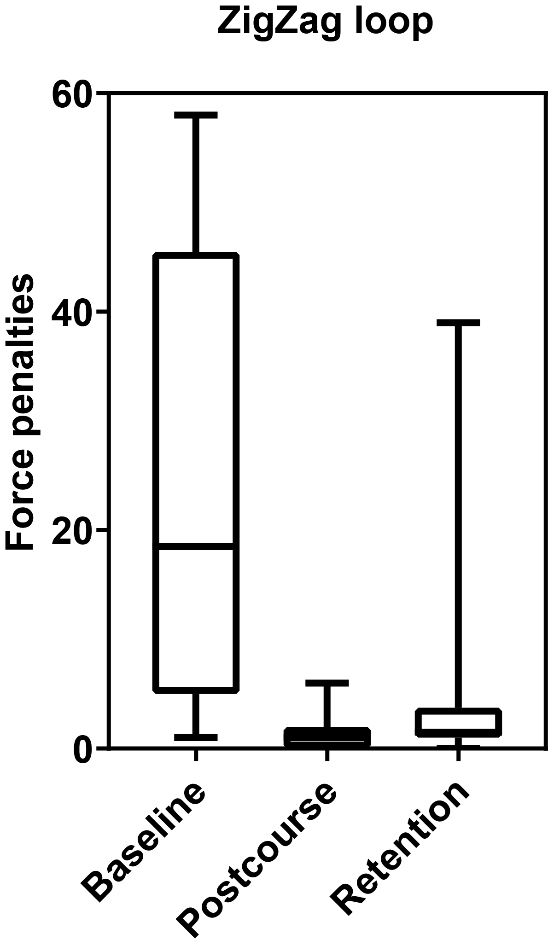

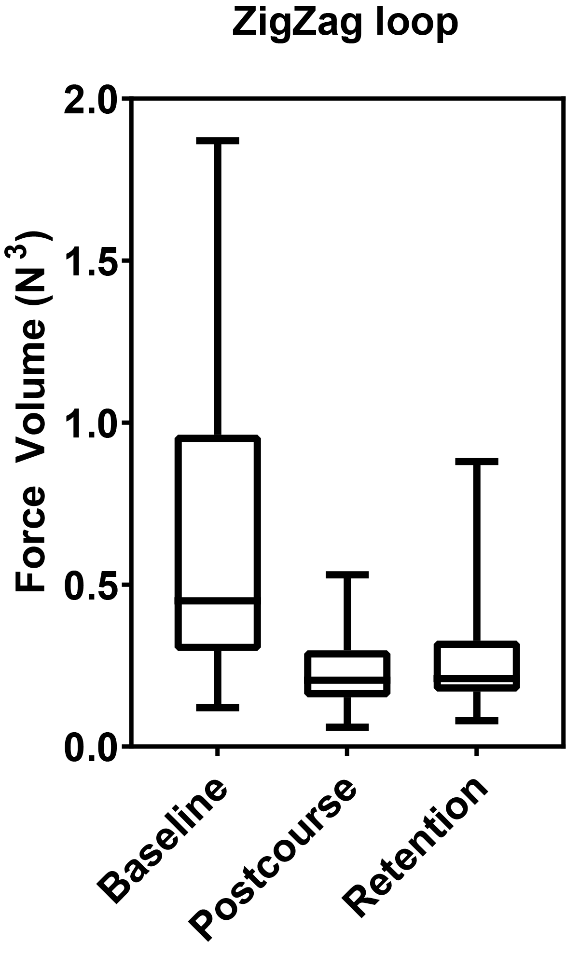

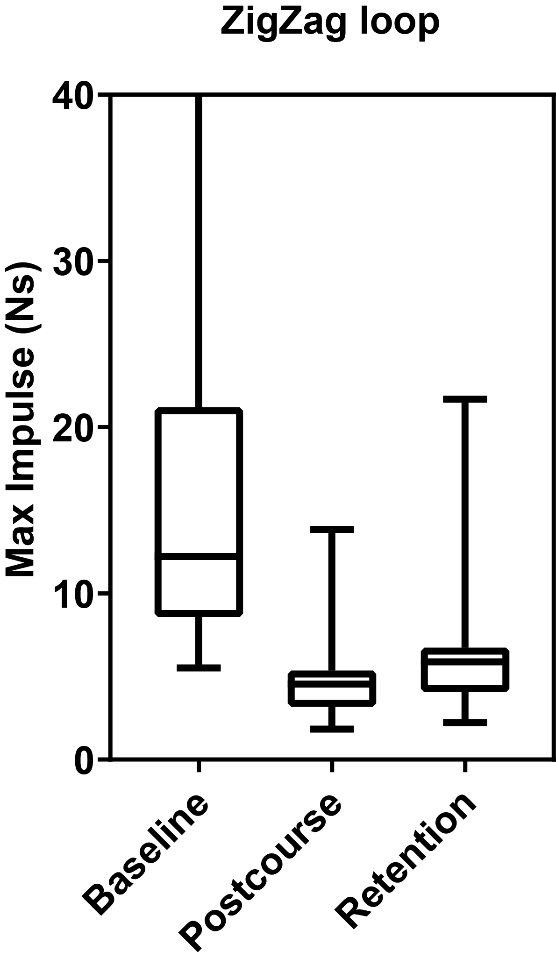


**Graph B3**

*Post and Sleeve and ZigZag loop boxplots of the force, path length and time measurements*


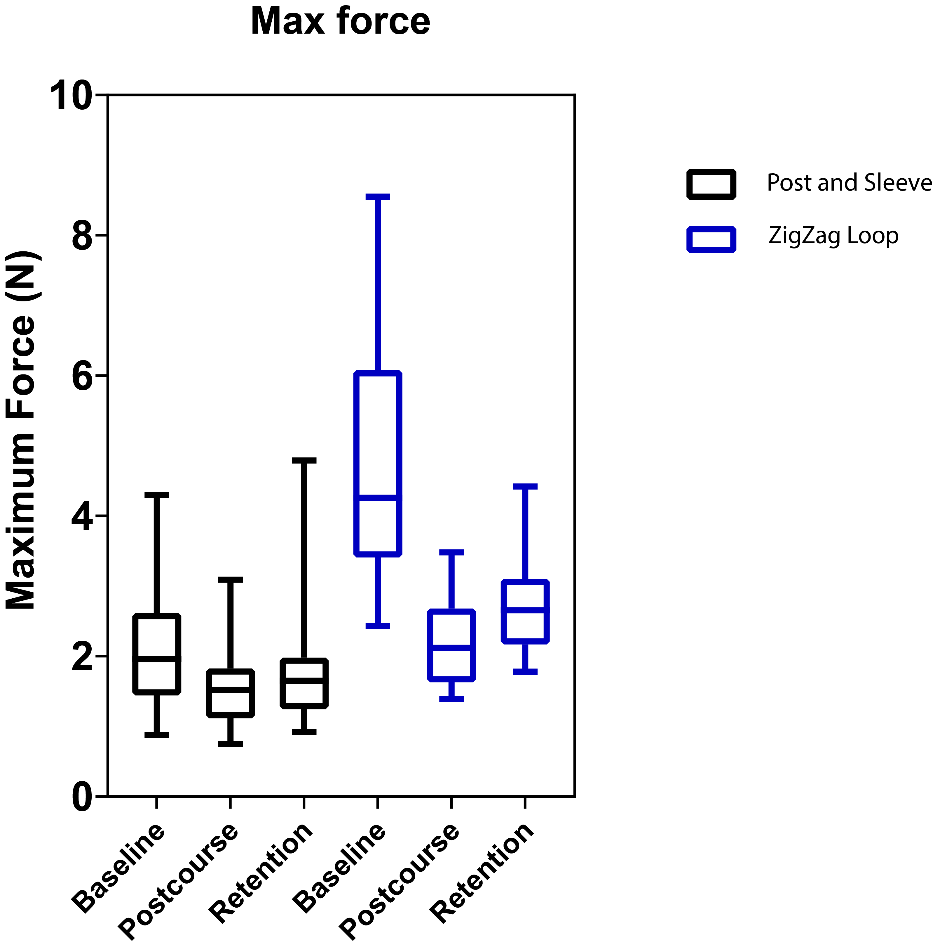


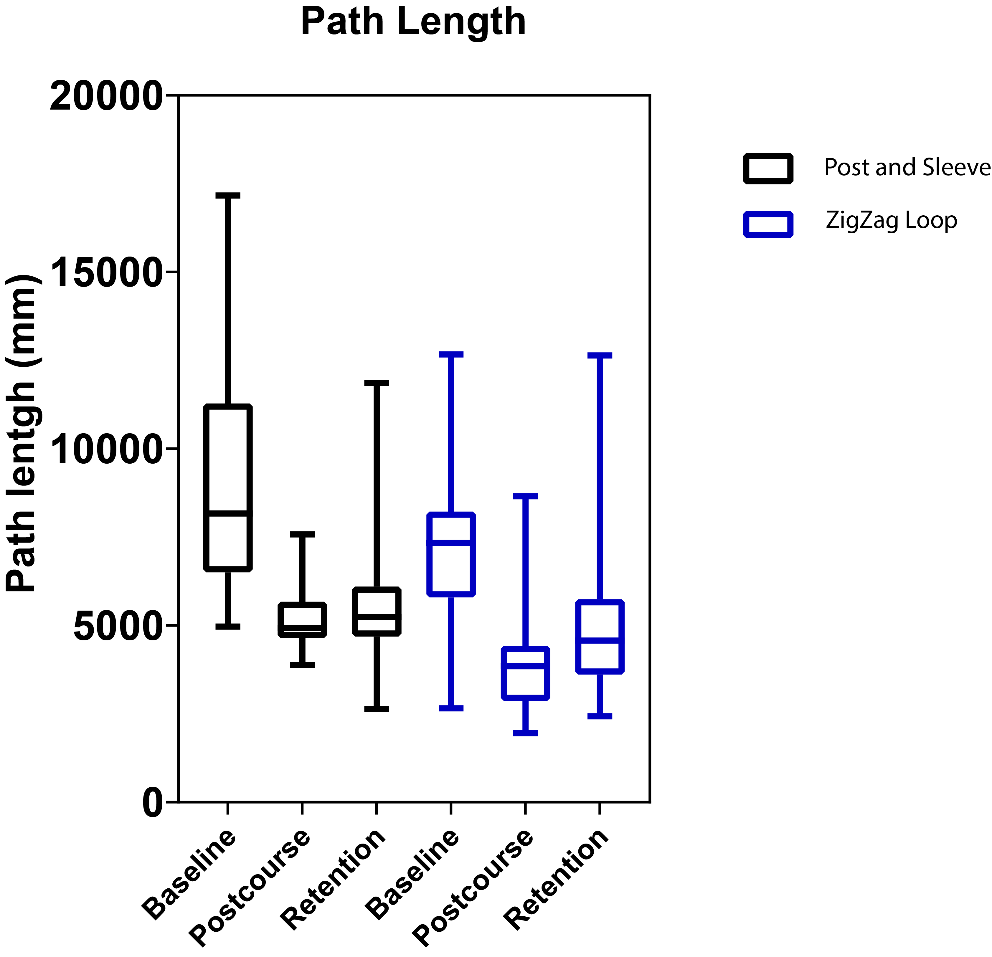


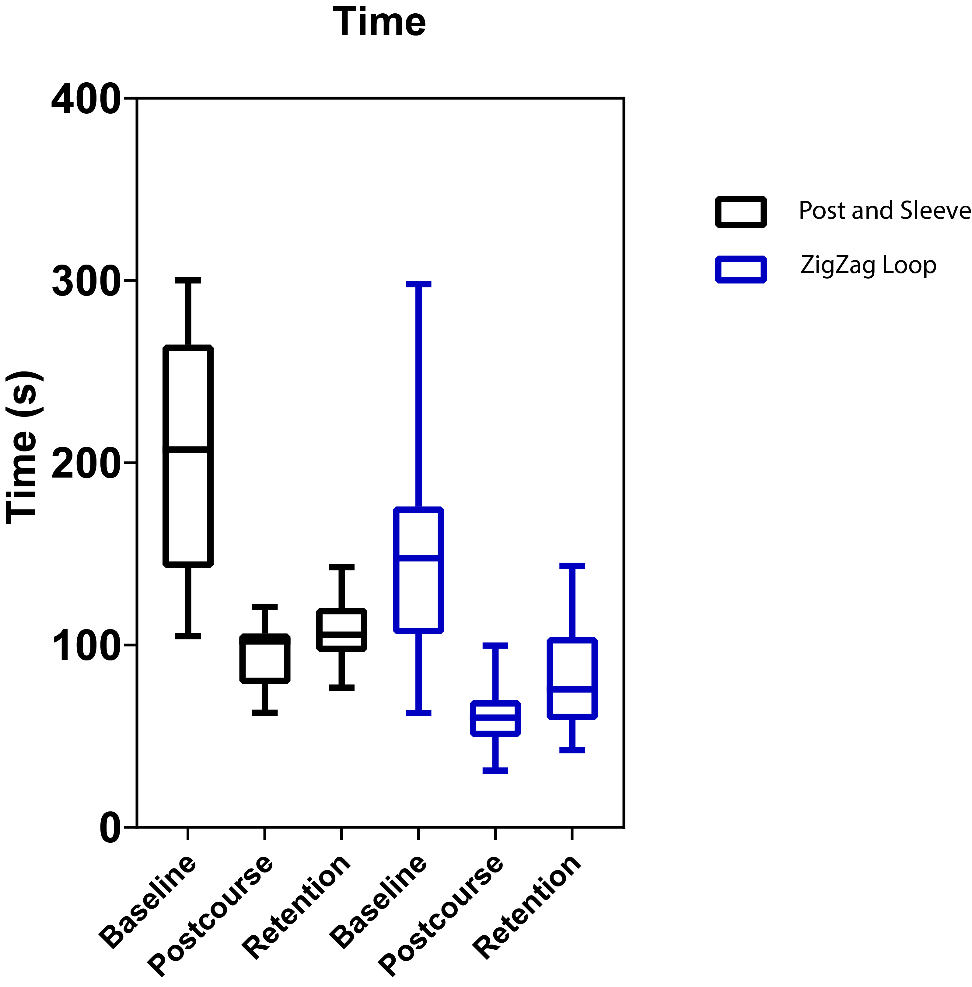

Supplement: Supplementary file 1 [file js9-109-0723-s001.docx]
